# Prognostic significance and expression pattern of glucose related genes in breast cancer: A comprehensive computational biology approach

**DOI:** 10.1016/j.sjbs.2023.103896

**Published:** 2023-12-10

**Authors:** Bader Alshehri

**Affiliations:** Department of Medical Laboratory Sciences, College of Applied Medical Sciences, Majmaah University, Almajmaah 11952, Saudi Arabia

**Keywords:** Breast cancer, Warburg effect, Deregulation, Prognosis, Survival analysis, Bioinformatics, Glucose metabolism

## Abstract

Breast cancer (BC) is the most common type of malignancy globally and the main reason why women die from tumours. The Warburg effect, a characteristic of tumor, describes how most solid tumour cells acclimatize to their diverse surroundings by relying heavily on aerobic glycolysis for production of energy. In addition to producing key metabolic intermediates that are crucial for the production of macromolecules, which enable cancer cell division, invasiveness, and drug resistance, the transformed energy metabolism also supplies tumor cells with ATP for cellular energy. Here, we evaluated the expression profile, prognostic significance, and clinical relevance of glucose-related genes in BC using a bioinformatic approach. To clarify the significance of glucose-related genes in the development of breast tumours, we also performed a functional enrichment investigation of deregulated genes using the STRING and KEGG portal. The study depicted that of the 61 genes examined, 8 genes had a fold change </=± 1.5, that is, ADH1C, ADH4, ALDH1A3, ALDOC, FBP1, PCK1, PFKFB1, PFKFB3. Among the highly deregulated genes, ADH1C showed a fold change of −6.669. These deregulated genes were associated with poor prognosis. The study signifies that glucose related genes are highly dysregulated in breast cancer. Deregulation of glucose related genes is linked with a poor prognosis in BC individuals. Thus, targeting glucose related genes will provide an effective treatment approach for BC individuals.

## Highlights

• This study evaluated the expression profile, prognostic role, and clinical relevance of glucose-related genes in BC using a bioinformatic approach.
• The study depicted that the 8 genes out of 61 explored genes showed a fold change </=± 1.5, that is, ADH1C, ADH4, ALDH1A3, ALDOC, FBP1, PCK1, PFKFB1, PFKFB3.
• The study signifies that glucose related genes are highly dysregulated in breast cancer.
• Deregulation of glucose related genes is linked with a poor prognosis in BC individuals.
• Thus, targeting glucose related genes will provide an effective treatment approach for BC individuals.

## Graphical abstract

**Figure:**
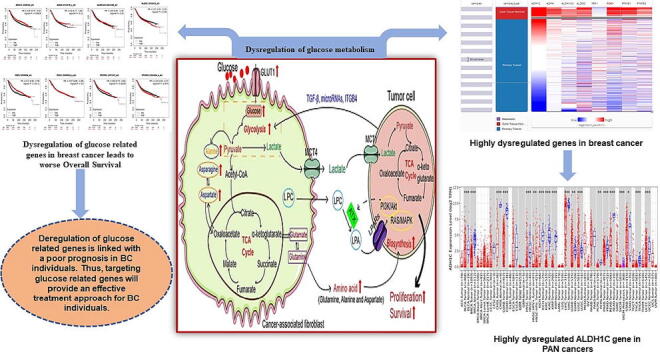

## 1 Introduction

BC is the most recurrent type of malignancy found in females, and it is the 2nd most common reason of mortality in females globally. With a predictable 2.3 million new cases, or 11.7 % of all tumor cases, BC in females have now beaten lung cancer as the most frequent form of cancer in 2020 (Sung et al., 2021, Mehraj et al., 2022, Mehraj et al., 2022). While mortality differed threefold for men and marginally for women, total incidence was 2–3 times higher for both sexes in transitioned compared to transitional countries. By 2040, the number of newly diagnosed BC patients is expected to increase by over 40 %, to about 3 million cases every year. Also, deaths from BC are set out to project more than 50 %, from 685,000 in 2020 to 1 million in 2040(Morgan et al., 2023). Even with improvements in early detection and treatment, BC remains the main factor in tumor-related fatalities. One of the main reason of treatment failures is the heterogeneity of breast cancer and distant metastases. Following diagnosis and primary tumour treatment, 20–30 % of breast cancer patients may develop metastases, and metastasis is thought to be responsible for 90–100 % of mortalities related to cancer (Lee and Djamgoz, 2018, Mehraj et al., 2022). Without metastasis, the 5 year OS rate for BC individuals is greater than eighty percent (Scully et al., 2012, Khan et al., 2022), but distant metastasis can dramatically lower this rate to only 25 % (Scully et al., 2012, Sofi et al., 2022). With more than twenty different subgroups that vary in genetics, appearance, and clinical behavior, BC is a highly diverse solid tumour. Breast cancer has been divided into lum A, lum B, HER2, and TNBC subtypes based on gene expression (Mir et al., 2022). TNBC, which makes up around 20 % of all instances of BC, is identified by the lack of the ER, and PR receptors as well as the overexpression of the HEGF-R2 or HER2 (Mir et al., 2022). Due to poor treatment response and the emergence of metastatic and recurrent malignancies, BC remains a challenge despite improvements in detection and therapy techniques (O’Reilly et al., 2015, Mehraj et al., 2021, Sofi et al., 2022). Early screening, or the early identification of a cancer with no symptoms in a population of individuals who seem to be in excellent health, and early diagnosis, or the early discovery of cancer symptoms are two independent but linked approaches that the WHO has identified to support early cancer detection (Qayoom and Bhat, 2020, Yousuf et al., 2022). Many BC individuals in low- and middle-income countries (LMICs) present or receive a final diagnosis of later-stage (locally progressed or metastatic) ailment (Martei et al., 2018, Mir and Mehraj, 2019). The ability to distinguish metabolic variations among BC molecular subtypes aids in adjusting tactics to target particular enzymes and transporters. Clinical trials benefit from metabolomic research, which broaden the definition of tumour classification beyond the current molecular subtypes. Patients with primary BC who had surgery exhibited metabolic variations among BC subtypes. Cappelletti V. et al. reported that for the metabolism of glucose, there are significant variations in the enzyme activity and pathways (Cappelletti et al., 2017).

It is commonly acknowledged that reprogramming of cellular energy metabolism is a cancer characteristic (O’Reilly et al., 2015, Mir et al., 2022, Shah, 2023). Even when there is oxygen, tumor cells exhibit an abnormal energy metabolism referred to as the Warburg effect, which involves substantially augmented rates of glucose uptake, glycolytic oxidation, and lactic acid generation (Khan et al., 2023). Cancer cells therefore require more glucose and are more sensitive to the mortality caused by glucose deprivation than normal cells. Therefore, glucose uptake inhibitors may be used as therapeutic targets in cancer (Barbosa and Martel, 2020, Qayoom et al., 2023). The requirement for glucose in tumor cells is larger than in normal cells because the energetic metabolic shift in these cells results in fewer ATP being produced per glucose molecule (Fadaka et al., 2017). Therefore, to meet their increased requirements for energy, biosynthesis, and redox, tumor cells depend on augmented rates of glucose uptake. Upregulation of glucose transporters, which is seen in the majority of tumor cells, is used to accommodate the cellular uptake of glucose at this higher pace (Szablewski, 2013). The activation of the pathways that regulate the expression of the enzymes in BC results in the overexpression of GLUTs and enzymes associated to glycolysis (Wu et al., 2020, Qayoom et al., 2022). Among the GLUT proteins, GLUT1 and GLUT3 have been chiefly reported to be involved in many cancer, such as colorectal carcinoma, leukemia, breast cancer, and glioblastoma. Increased expressions of GLUT1 and GLUT3 are also linked with worse clinical outcomes in patients with glioblastoma and colorectal carcinoma (Cosset et al., 2017, Dai et al., 2020). PKM2 is another glycolytic enzyme, the higher expression of which converts phosphoenolpyruvate to produce pyruvate and ATP in tumor cells like pancreatic cancer cells (James et al., 2020). Furthermore, PFKP was reported to be associated with epithelial–mesenchymal transition in BC cells, and its overexpression is a poor prognostic factor for BC patients with BRCA1 deficiency (Kim et al., 2017). The research reveals that breast cancer exhibits varied glycolytic activity depending on the kind of tumour cell, which is an example of metabolic intratumoral heterogeneity (Yu et al., 2021, Mir et al., 2022). BC has metabolic variations between primary and metastatic forms, and it utilizes a variety of metabolic pathways rather than depending solely on one (Mehraj et al., 2022). In contrast to non-metastatic 67NR BC cells, extremely metastatic 4 T1 cells exhibit enhanced glycolysis and OXPHOS (Dupuy et al., 2015, Khan et al., 2023). The most frequent metastatic locations are the liver, lung, bone, and brain, which display distinct metabolic characteristics because of their various microenvironments (Chen et al., 2018). In contrast to bone and lung metastatic BC, liver metastatic BC exhibits higher glycolytic pathways, while brain metastatic BC exhibits greater PPP and glycolysis compared to bone metastatic BC (Hanahan and Weinberg, 2011, Khan et al., 2022).

From the above its very clear that glucose is the primary energy source for mammalian cells. Tumor cells typically rely on aerobic glycolysis to meet their biosynthetic need for energy, lipids, nucleotides, and amino acids, thus promoting cell survival and proliferation. Inhibitors of glucose metabolism can also be thought of as a combination therapy with traditional treatments as glucose metabolism is linked with chemoresistance or targeted treatments in BC (Abdel-Wahab et al., 2019). In recent years, various mutated or dysregulated transcription factor targets were found in tumors. They are a specific class of anticancer drug targets that modulate aberrant gene expression, including genes related to glucose metabolism and metastasis.

In existing study, we have discovered the role of glucose deregulation in BC. We have analyzed the expression profile, their survival analysis, and their correlation with various clinico pathological features, protein -protein, gene -gene interaction, gene ontology and KEGG enrichment of various glucose related genes in breast cancer. The uniqueness of this work lies in the fact that we have used bioinformatics to comprehensively analyze their expression patterns and their relationship with survival in breast cancer metastasis and provide a new strategy for the treatment of breast cancer. The study overall, signified the importance of deregulated genes in BC progression.

## 2 Methodology

### 2.1 Differentially expressed genes regulating glucose metabolism in BC

The expression profile of genes related to glucose in BC was assessed by means of the UCSC XENA online portal, and a heat map was developed. Additionally, the fold change in the gene expression associated to glucose in breast cancer was assessed using the online data mining platform Gepia2 (https://gepia2.cancer-pku.cn/) (Tang et al., 2017). This online database contains 1085 BCE samples and 291 GTEx samples. The database has got several datasets and data hubs and can be used for several other purposes like survival analysis etc.

### 2.2 Expression analysis of dysregulated glucose related genes in PAN cancer and different subclasses of BC

We also studied the expression profile of vastly dysregulated glucose-related genes in several cancers using TIMER 2 portal (https://timer.cistrome.org/). TIMER, an online web resource assesses the expression profile and immune infiltrates in several forms of cancers (Li et al., 2020). Also, the expression in several subclasses of BC patients was studied by assessing the UALCAN portal, an online resource for learning cancer OMICS data (Chandrashekar et al., 2017, Chandrashekar et al., 2017).

### 2.3 Kaplan -Meier plotter

An online resource known as KM plotter (https://kmplot.com) provides details on gene expression and the survival of BC individuals (Györffy et al., 2010). RFS data for 4929 individuals and OS data for 1879 individuals are included in the mRNA gene chip breast cancer dataset of the KM Plotter. Based on the gene's median expression, the individuals were grouped into 2 cohorts. KM-survival curves were used to examine the impact of these dysregulated genes on RFS and OS of the 2 designated groups, and the KM plotter was used to determine the HR intervals and the log-rank P-value.

### 2.4 bc-GenEXMiner

(https://bcgenex.centregauducheau.fr/BC-GEM) Breast cancer gene-expression miner' (bc-GenExMiner) is a breast cancer-associated web portal (https://bcgenex.ico.unicancer.fr). bc Gene-Expression Miner V4.5 (bC-GenEXminerv4.5) is a web-based portal for annotated BC transcriptome data (Jézéquel et al., 2012, Jezequel et al., 2013).

The statistical analyses are grouped in three modules: “correlation”, “expression” and “prognostic”. bc-GenEXMiner was assessed to explore the relationships between the expression levels of glucose-related genes and several clinical features of BC individuals, including hormonal status, nodal profile, age group in breast cancer patients.

### 2.5 Gene-gene and protein-protein interaction analysis

We assesed the internet resource GENEMANIA to estimate the role and connection of a subset of glucose-related genes for gene-gene interaction study (https://genemania.org/) (Warde-Farley et al., 2010). With a confidence score value of 0.7, a PPI grid of deregulated glucose-related genes was formed by means of the STRING (https://stringdb.org). STRING is a biological database formed to develop and assess the functional interactions between the proteins (Szklarczyk et al., 2015). By means of the Cytoscape programme (v 3.8.2), the network was also studied and envisaged (Smoot et al., 2011). To obtain the noteworthy modules in the said PPI network, the MCODE plug-in for the Cytoscape software was used (Bader and Hogue, 2003). The HUB proteins with the topmost 13 degrees in the PPI web were gained via the cytohubba plugin (Chin et al., 2014).

### 2.6 Functional enhancement of highly deregulated glucose genes

The KEGG (Kyoto Encyclopedia of Genes and Genomes) pathway enrichment study and the gene ontology function were explored using the Mayan lab online resource (https://maayanlab.cloud/Enrichr/) (Kuleshov et al., 2016). It is an integrative web-based and mobile software application that includes new gene-set libraries, an alternative approach to rank enriched terms, and various interactive visualization approaches to display enrichment results using the JavaScript library, Data Driven Documents (D3). GO and KEGG keywords that have an FDR less than 0.05 were considered to be important pathways and functions.

## 3 Results

### 3.1 Differential gene expression of glucose metabolism genes in breast cancer patients

**Fig. 1:**
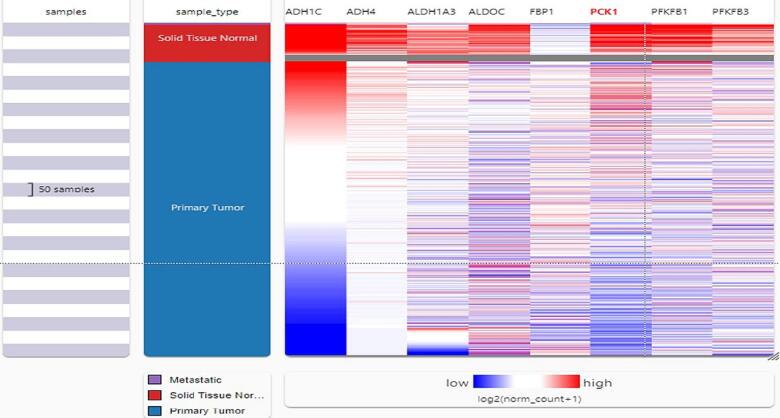
heat map of various deregulated glucose related genes in breast cancer using UCSC XENA browser. The highest downregulation was seen in case of ADH1C.

Using the Gepia2 web site, a differential gene expression analysis was analyzed on the TCGA BrCA data set. The Glucose related genes were extremely dysregulated in BC individuals. Also, out of the 61 screened genes, 8 genes that is, ADH1C, ADH4, ALDH1A3, ALDOC, FBP1, PCK1, PFKFB1, and PFKFB3 show log2 fold >±1. **(**Table1**).** ADH1C was downregulated with log2 fold change of −6,669. we further develop a heat map of these dysregulated genes involved in glucose metabolism using USCS XENA portal **(**Fig. 1**).**

**Table 1:** Log 2-fold change of several glucose related genes in breast cancer.

*GENE symbols*	GENE ID	Median (tumor)	Median (NORMAL)	Log2 fold change	Adjp
ACSS2	ENSG00000154930.14	17.500	14.560	0.250	5.86e-3
ADH1A	ENSG00000131069.19	20.990	56.759	−1.393	4.16e-200
ADH1B	ENSG00000187758.7	0.020	1.050	−1.007	6.82e-228
ADH1C	ENSG00000196616.12	4.620	570.703	**−6.669**	3.50e-250
ADH4	ENSG00000248144.5	0.460	16.130	**−3.553**	2.32e-310
ADH5	ENSG00000197894.10	83.742	128.863	−0.616	6.11e-77
ADH6	ENSG00000172955.17	0.050	0.450	−0.466	6.31e-166
AKR1A1	ENSG00000117448.13	151.439	88.849	0.763	2.59e-110
ALDH1A3	ENSG00000184254.16	7.120	22.330	**−1.523**	5.42e-56
ALDH1B1	ENSG00000137124.6	17.830	9.280	0.873	6.01e-71
ALDH2	ENSG00000111275.12	68.161	174.864	−1.346	5.87e-75
ALDH3A1	ENSG00000108602.17	0.220	2.050	−1.322	5.83e-78
ALDH3A2	ENSG00000072210.18	30.840	37.681	−0.281	1.52e-7
ALDH3B1	ENSG00000006534.15	6.050	4.980	0.238	6.68e-7
ALDH3B2	ENSG00000132746.14	30.250	12.760	1.183	8.45e-24
ALDH7A1	ENSG00000164904.15	74.403	55.180	0.425	1.28e-13
ALDOA	ENSG00000149925.16	1645.715	831.515	0.984	2.03e-83
ALDOB	ENSG00000136872.17	0.050	0.200	−0.193	1.05e-20
ALDOC	ENSG00000109107.13	9.200	35.199	**−1.827**	1.05e-90
BPGM	ENSG00000172331.11	10.720	9.895	0.105	7.42e-3
DLAT	ENSG00000150768.15	18.621	16.250	0.186	5.30e-3
					
ENO1	ENSG00000074800.13	562.417	457.712	0.297	1.13e-17
ENO2	ENSG00000111674.8	19.510	14.930	0.365	1.73e-3
ENO3	ENSG00000108515.17	5.220	9.840	−0.801	2.54e-20
FBP1	ENSG00000165140.9	74.578	22.011	**1.716**	1.57e-39
FBP2	ENSG00000130957.4	0.030	0.070	−0.055	2.45e-5
G6PC1					
G6PC2	ENSG00000152254.10	0.000	0.040	−0.057	4.51e-27
GALM	ENSG00000143891.16	9.200	7.860	0.203	4.12e-5
GAPDH	ENSG00000111640.14	2525.756	1147.437	1.138	2.88e-132
GAPDHS					
GCK	ENSG00000106633.15	0.130	0.510	−0.418	5.29e-92
GPI	ENSG00000105220.14	188.079	133.241	0.494	1.72e-35
HK1	ENSG00000156515.21	79.970	45.759	0.792	5.02e-62
HK2	ENSG00000159399.9	21.290	14.370	0.536	1.84e-15
HK3	ENSG00000160883.10	1.810	2.310	−0.236	3.66e-3
HKDC1					
LDHA	ENSG00000134333.13	566.918	394.750	0.521	1.96e-26
LDHAL6A					
LDHAL6B	ENSG00000171989.5	0.030	0.100	−0.095	1.27e-79
LDHB	ENSG00000111716.12	118.118	320.170	−1.431	2.77e-50
LDHC	ENSG00000166796.11	0.380	0.200	0.202	6.35e-9
PANK1	ENSG00000152782.16	2.930	1.740	0.520	4.38e-19
PCK1	ENSG00000124253.10	0.160	13.500	**−3.644**	2.85e-230
PCK2	ENSG00000100889.11	34.479	20.180	0.744	5.90e-41
PDHA1	ENSG00000131828.13	88.480	103.881	−0.229	2.95e-7
PDHA2					
PDHB	ENSG00000168291.12	59.779	47.320	0.331	2.77e-19
PFKFB1	ENSG00000158571.10	0.470	4.940	**−2.015**	6.99e-174
PFKFB2	ENSG00000123836.14	5.700	3.760	0.493	3.15e-14
PFKFB3	ENSG00000170525.18	29.331	91.670	**−1.611**	6.09e-88
PFKFB4	ENSG00000114268.11	4.660	2.110	0.864	7.44e-95
PFKL					
PFKM	ENSG00000152556.15	26.051	28.660	−0.133	7.82e-3
PFKP					
PGAM1	ENSG00000171314.8	168.452	118.923	0.499	2.41e-51
PGAM2	ENSG00000164708.5	0.520	1.310	−0.604	1.67e-8
PGAM4	ENSG00000226784.2	0.150	0.100	0.064	1.32e-7
PGK1	ENSG00000102144.13	345.489	191.300	0.849	8.74e-71
PGK2					
PGM1	ENSG00000079739.15	31.380	47.399	−0.580	7.03e-36
PGM2	ENSG00000169299.13	18.970	12.710	0.543	6.83e-28
PKLR					
PKM	ENSG00000067225.17	601.032	270.052	1.151	1.60e-141
SLC2A2					
TPI1	ENSG00000111669.14	476.554	271.272	0.811	1.52e-92

**Fig. 2:**
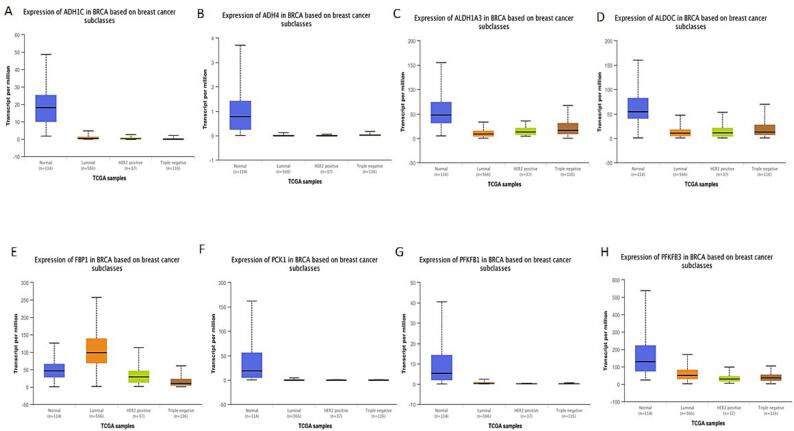
Expression pattern of extremely deregulated glucose related genes among several BC subclasses using UALCAN database. FBP1, shows high expression in Luminal BC as compared to the normal patients. Whereas, very low expression of ADH1C, ADH4, ALDH1A3, ALDOC, PCK1, PFKFB1, and PFKFB3 was seen in all BC subtypes including Luminal when compared to the normal patients.

**Fig. 3:**
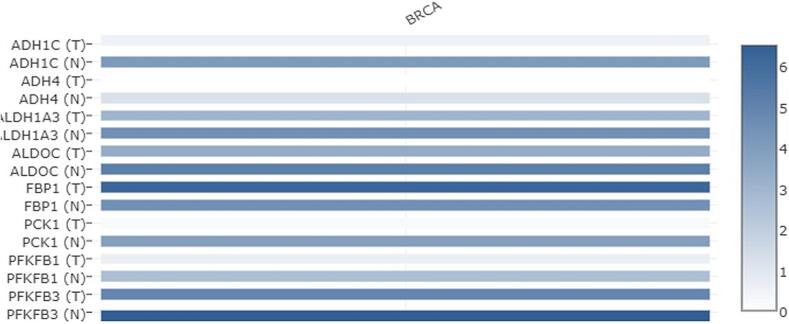
Expression profile of highly deregulated genes in different malignancies examined through Timer database. ADH1C, ADH4, ALDH1A3, ALDOC, FBP1, PCK1, PFKFB1, and PFKFB3 show downregulation in breast carcinoma in contrast to normal breast tissue. All these genes are downregulated in tumor cells as compared to the normal ones.

The heat map showed that all the 8-dysregulated glucose related genes are highly downregulated in BC unlike their normal tissues**.** Also, we explore the expression of highly deregulated glucose related genes among several BC subclasses using UALCAN database. FBP1, shows high expression in Luminal BC as compared to the normal patients. Whereas, very low expression of ADH1C, ADH4, ALDH1A3, ALDOC, PCK1, PFKFB1, and PFKFB3 was seen in all BC subtypes including Luminal when compared to the normal patients **(**Fig. 2**).** Furthermore, the expression of highly deregulated genes in different malignancies examined through Timer database also reflected that these genes are downregulated in tumor individuals in contrast to normal ones **(**Fig. 3**)**.

### 3.2 Deregulation of ADH1C across several cancers

**Fig. 4:**
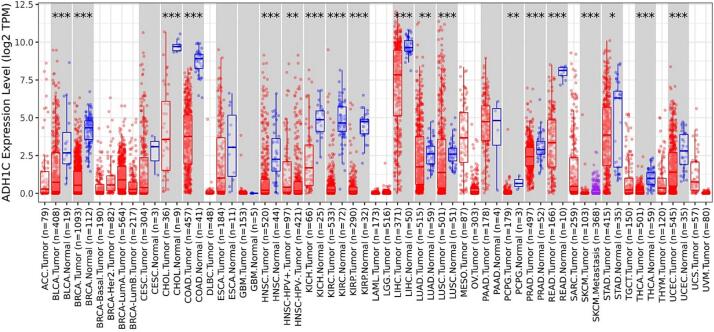
Deregulation of ADH1C across several cancers. ADH1C expression is downregulated in several cancers including Breast cancer (P < 0.01).

The analysis of GEPIA 2 revealed that among the 8-downregulated glucose related genes, ADH1C shows highest downregulation as also reflected by the fold change **(**Table 1**).** ADH1C was downregulated with log2 fold change of −6,669. Next, the TIMER exploration of the ADH1C was used to analyse its expression. The results showed ADH1C deregulation across all TCGA datasets, as shown in the in Fig. 4**.** The results revealed that ADH1C expression is downregulated in several cancers including BC (P < 0.01).

### 3.3 Deregulation of glucose related genes is linked with a worse prognosis in BC individuals

The KM Plotter, which comprises gene expression profile information as well as the survival analysis of BC individuals, was assessed to explore the survival analysis of extremely dysregulated glucose related genes; ADH1C, ADH4, ALDH1A3, ALDOC, FBP1, PCK1, PFKFB1, PFKFB3. BC individuals were separated into 2 groups, referred to as low and high expression groups based on median expression. The overall survival revealed that increase in ALDH1C, ADH4, and PFKFB3 correlates with high survival probability as compared to the ALDH1A3, FBP1 and PCK1 which showed higher survival probability at lower levels of their expression (P < 0.05) **(**Fig. 5A**).** Rest of the genes does not show any significant correlation between OS and survival probability, and their respective p value was not also statistically significant. The RFS graphs depicted that high levels of ADH4 and FBP1 show higher RFS compared to ALDOC and PFKFB3 which show higher RFS at their low levels of mRNA expression (P < 0.05) **(**Fig. 5B**).**

**Fig. 5A:**
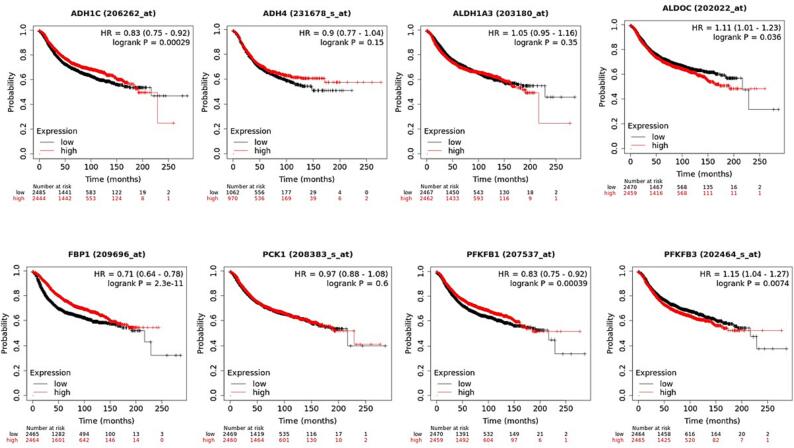
The overall survival revealed that increase in ADH1C, ADH4, FBP1 and PFKFB1 correlates with high survival probability as compared to the ALDH1A3, ALDOC, PFKFB3 and PCK1 which showed higher survival probability at lower levels of their expression (P < 0.05).

**Fig. 5B:**
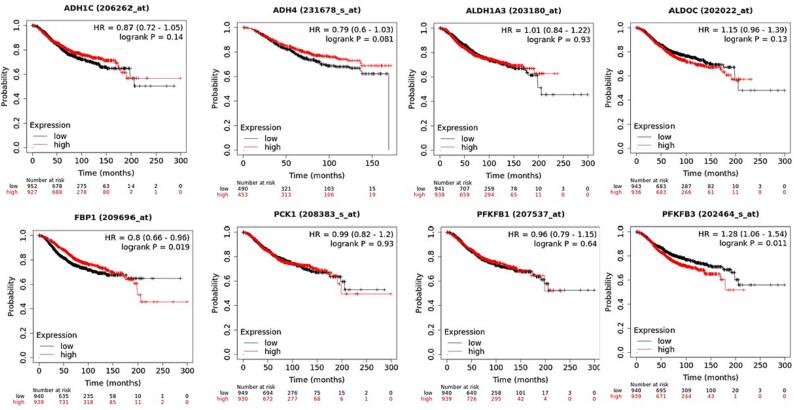
The RFS graphs reflected that high levels of ADH4 and FBP1 show higher RFS compared toPFKFB3 which show higher RFS at their low levels of mRNA expression (P < 0.05). The rest of the genes doesnot show much significance interms of p value.

### 3.4 Association of deregulated glucose related genes with clinical-physiological features in BC

**Fig. 6:**
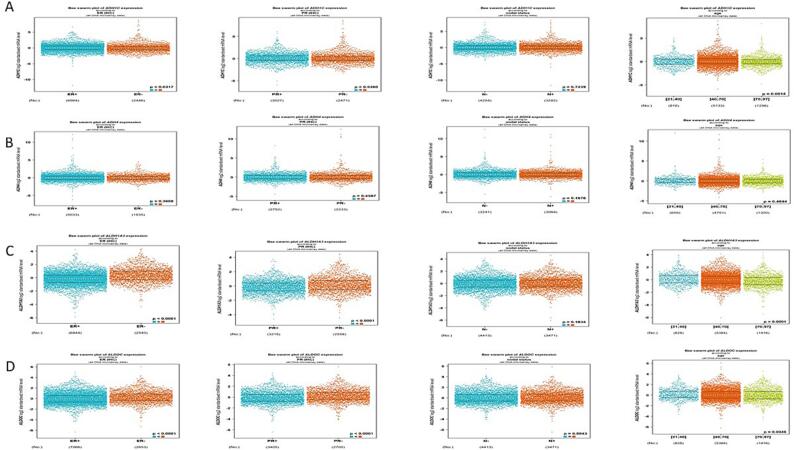

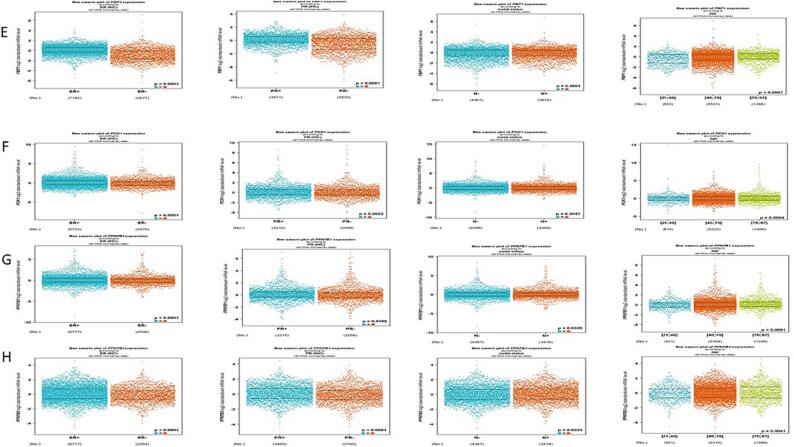
Correlation of deregulated glucose related genes with clinical-physiological characteristics in BC. A)ADH1C, B) ADH4, C) ALDH1A3, D) ALDOC Correlation of deregulated glucose related genes with clinical-physiological characteristics in BC. E) FBP1, F) PCK1, G) PFKFB1, H) PFKFB3.

The bc-GenEXMiner portal was utilized to correlate the link among dysregulated Glucose related genes ADH1C, ADH4, ALDH1A3, ALDOC, FBP1, PCK1, PFKFB1, PFKFB3 and several clinical-pathological characteristics in BC individuals. ADHIC expression was pointedly elevated in BC positive for ER (p = 0.0317). The expression of ADH1C in PR + BC didn’t vary substantially (P = 0.5360). In terms of nodal profile, it was analyzed that the expression of ADH1C does not vary among the N + and N- groups (P = 0.7239). Additionally, the expression of ADH1C was highest in the age group of 40–70 (P = 0.0014) **(**Fig. 6**A).**

ADH4 expression, however, did not vary substantially from ER, PR, nodal profile. ADH4 on the other hand, was shown to be significantly very high in the age set of 40–70 years of breast cancer patients **(**Fig. 6**B).**

ALDH1A3 expression was pointedly higher in ER-ve and PR-ve BC individuals (P < 0.0001). Also, the expression of ALDHIA3 did not vary substantially from nodal profile (P = 0.1834). ALDH1A3 expression was highest in the age group of 40–70 having p value < 0.001 **(**Fig. 6**C).**

ALDOC expression was pointedly higher in ER-ve and PR-ve breast cancer individuals. (P < 0.0001). The expression in terms of nodal profile doesn’t vary substantially and was higher in the age period of 40–70 (P value 0.803 and 0.0046 respectively) **(**Fig. 6**D).**

FBP1 expression was pointedly augmented in ER + ve and PR + ve BC individuals (P < 0.0001). In terms of nodal profile its expression was almost equal in both the groups (P = 0.4801). Similarly, the expression of FBP1 was highest in the age group of 40–70 (P < 0.0001) **(**Fig. 6**E)**.

PCK1 expression was pointedly augmented in ER and PR + ve BC individuals (P value < 0.0001 and0.0053 respectively). In relation to nodal profile there was no significant alteration in their PCK1 expression (P = 0.3047). Also, the expression was maximum in the age group of 40–70 years (p = 0.0004) **(**Fig. 6**F).**

PFKFB1 and PFKFB2 expression was pointedly higher in ER and PR + ve BC individuals (P values = 0.0001). Also, the expression of PFKFB1 and PFKFB2 was enhanced in the age group of 40–70 (p value < 0.0001 and 0.041respectively) **(**Fig. 6**G &H).**

### 3.5 Gene ontology and pathway enrichment analysis

**Fig. 7:**
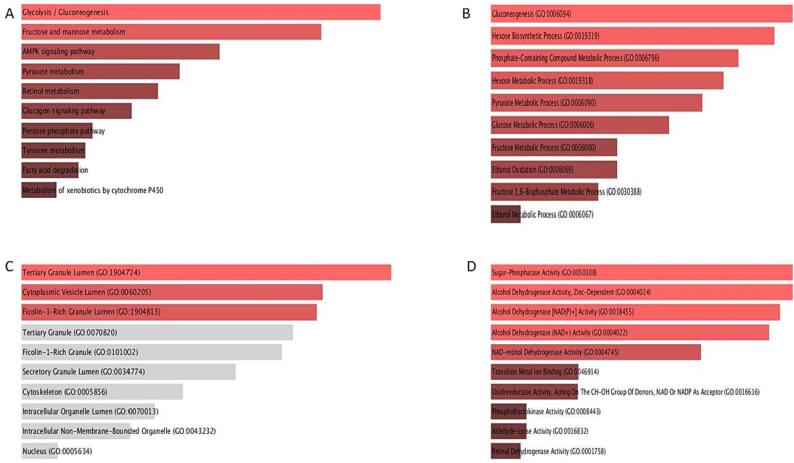
(A-D). Gene ontology and pathway analysis of deregulated glucose related genes. A) KEGG pathway, B) Biological process, C) Cellular compartment, D) Molecular function.

We analyzed GO and pathway enrichments linked to glucose metabolism, by using the Enrichr portal. KEGG pathway depicted that the 8 glucose related genes are highly enriched in the processes like glycolysis, fructose and mannose metabolism, AMPK pathway, pyruvate metabolism, glucagon signalling pathway etc **(**Fig. 7**A)**. The GO of these glucose genes were pointedly augmented in biological processes. The enhancement was seen in these 8 genes in terms of gluconeogenesis, hexose biosynthetic process, phosphate-containing compound metabolic process, pyruvate metabolic process, ethanol oxidation etc. **(**Fig. 7**B).** The glucose-related genes in terms of cellular CC reflected enrichment in Ficolin-1-rich granule lumen, cytoplasmic vesicle lumen, tertiary granule lumen etc**. (**Fig. 7**C).** The proposed MF, in the molecular function group, was chiefly enriched in were enhanced in the functions like sugar-phosphatase activity, alcohol dehydrogenase activity, NAD-retinol dehydrogenase activity, transition metal ion binding, oxidoreductase activity, phosphofructokinase activity etc. **(**Fig. 7**D).**

### 3.6 PPI and GGI networks of dysregulated glucose related genes

**Fig. 8:**
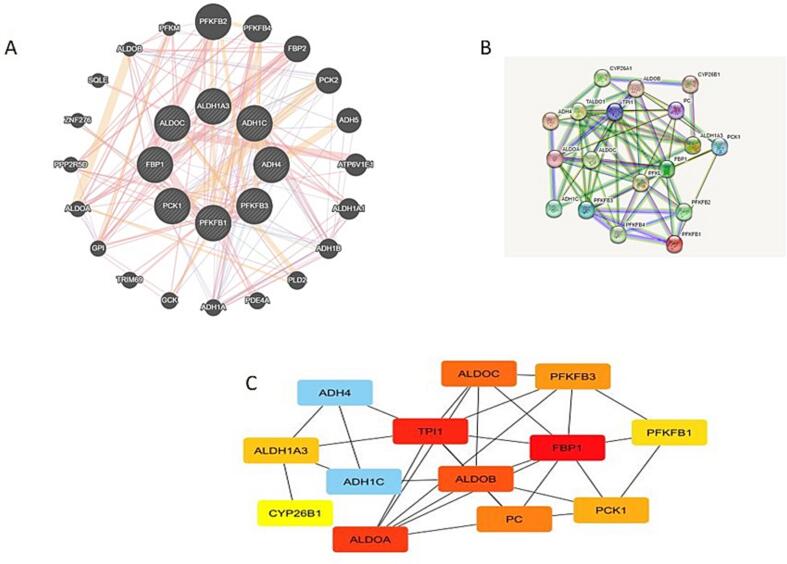
**A&B)** PPI and GGI networks of dysregulated glucose related genes using String and GENEMANIA database. C) Top hub genes of the glucose dysregulated gene network in breast cancer.

We further analyzed gene-gene interactions of deregulated glucose related genes in breast cancer, and it was found that deregulated glucose related genes show high interactions with PFKFB2, PFKFB4, FBP2, PCK2, ADH5 and other genes involved in glucose metabolism **(**Fig. 8**A).** The PPI grid was developed by employing STRING and the confidence score value of the network was > 0.7. The developed grid had 13 nodes and 31 edges, with an average node degree value of 4.77 and a PPI enrichment p-value of 9.71e-13. Furthermore, using the cytohubba plugin, the top 13 hub-genes of the web, based on degree score were recognized by employing Cytohubba as shown in Fig. 8**B.** Besides the above-mentioned genes, the other genes comprised TPI1, ALDOB, PC, CYP26B1, and ALDOA. The MCODE plug-in of the Cytoscape software was investigated to attain the most central part of the PPI network **(**Fig. 8**C). The top hub genes include; ADH4, ALDOC, PFKFB3, TP11, ALDOB, PC, ALDOA, ADH1C, CYP26B1, ALDH1A3, FBP1, PCK1, and PFKFB1**.

## 4 Discussion

According to the GLOBOCAN 2020, BC is the most widespread type of malignancy in females globally, representing almost twenty five percent of all malignancies in women. It has now exceeded lung cancer as the foremost reason of global cancer prevalence in 2020, with a predictable 2.3 million new reports, accounting 11.7 % of all cancer reports (Sung et al., 2021, Mir et al., 2023). Although improvements in early identification and therapy have increased the OS and RFS of BC individuals, there is an urgent need to pay attention to the emergence of metastatic and resistant cancers. (Benson and Jatoi, 2012, Anastasiadi et al., 2017). Since the early 1990 s, screening initiatives and adjuvant chemotherapy have considerably improved the survival, disease-free survival, and mortality rates of BC patients, improving their prognosis (Han et al., 2004, Qayoom et al., 2021). The incidence, death rates, and treatment-related morbidities of this disease all need to be reduced, thus work must to be done in this area. Finding new molecular targets and improving lead drugs are therefore top priorities in breast cancer research. The mechanisms beneath the drugs' modulatory impact on glucose uptake include direct effects on the transporter, suppression of transporter gene expression or protein synthesis, impairment of the transporter's membrane insertion, and manipulation of redox balance (Khan et al., 2023). Also, a decrease in the activity of glycolytic enzymes or signalling pathways may have a secondary impact on a substance's impact on glucose uptake. It must be mentioned, nonetheless, that for the majority of the chemicals discussed, the mechanisms behind their modulating effect on the uptake of glucose by BC cells have not been studied. Therefore, more study is required in this area. Improved understanding of the mechanisms that can affect glucose transporter activity in tumor cells may present novel treatment options for breast cancer.

The present study revealed some important observation related to the glucose related genes. Differential gene expression analysis revealed that Glucose related genes were dysregulated in BC Patients. out of the 61 screened genes, 8 genes that is, ADH1C, ADH4, ALDH1A3, ALDOC, FBP1, PCK1, PFKFB1, and PFKFB3 show log2 fold >±1**. (**Table1**).** ADH1C was downregulated with log2 fold change of −6.669. The heat map showed that all the 8-dysregulated glucose related genes are highly downregulated in BC as compared to their normal tissues **(**Fig. 1**).** The expression pattern of extremely deregulated glucose related genes across several BC subclasses using UALCAN database showed that very low expression of ADH1C, ADH4, ALDH1A3, ALDOC, PCK1, PFKFB1, and PFKFB3 in all BC subtypes including Luminal when compared to the normal patients **(**Fig. 2**).**

The overall survival revealed that increase in ALDH1C, ADH4, and PFKFB3 correlates with high survival probability as compared to the ALDH1A3, FBP1 and PCK1 which showed higher survival probability at lower levels of their expression (P < 0.05) (Fig. 5A). Rest of the genes does not show any significant correlation between OS and survival probability. The RFS graphs depicted that augmented levels of ADH4 and FBP1 show higher RFS compared to ALDOC and PFKFB3 which show higher RFS at their low levels of mRNA expression (P < 0.05) **(**Fig. 5B**).** Additionally, these deregulated genes show correlation with clinical physiological features in breast cancer individuals. The results reflected that ADHIC expression was pointedly elevated in BC positive for ER (p = 0.0317). Also, the expression of ADH1C was highest in the age group of 40–70 (P = 0.0014) **(**Fig. 6**A).**

ADH4 expression, however, did not vary considerably from ER, PR, nodal profile. ADH4 on the other hand, was shown to be significantly very high in the age group of 40–70 years of breast cancer patients (Fig. 6B). ALDH1A3 expression was significantly elevated in ER and PR -ve BC individuals (P < 0.0001). Also, the expression of ALDHIA3 did not vary substantially from nodal profile (P = 0.1834). ALDH1A3 expression was highest in the age group of 40–70 having p value < 0.001 (Fig. 6C). Furthermore, PFKFB1 and PFKFB2 expression was pointedly augmented in ER and PR + ve BC individuals, P values = 0.0001(Fig. 6G &H).

We also examined GO and pathways linked to glucose metabolism, by assessing the Enrichr portal. KEGG pathway analysis depicted that the said 8 glucose related genes are highly enriched in the processes like glycolysis, fructose and mannose metabolism, AMPK pathway, pyruvate metabolism, glucagon signalling pathway etc (Fig. 7A). GO analysis depicted that the said glucose genes were suggestively enriched in biological functions. An enrichment in the said genes in terms of gluconeogenesis, hexose biosynthetic process, phosphate-containing compound metabolic process, pyruvate metabolic process, ethanol oxidation etc. was also noticed (Fig. 7B).

The gene-gene interactions of deregulated glucose related genes in breast cancer, and it was found that deregulated glucose related genes show high interactions with PFKFB2, PFKFB4, FBP2, PCK2, ADH5 and other genes involved in glucose metabolism (Fig. 8A). Furthermore, by employing the cytohubba plugin, the topmost 13 hub-genes of the web, based on degree score were recognized as shown in Fig. 8B. Besides the above-mentioned genes, the other genes included TPI1, ALDOB, PC, CYP26B1, and ALDOA. The MCODE plug-in of the Cytoscape software was examined to get the most significant part of the PPI web (Fig. 7C). Overall, the study revealed that out of 61 screened genes, 8 genes show a fold change </=± 1.5, that is, ADH1C, ADH4, ALDH1A3, ALDOC, FBP1, PCK1, PFKFB1, PFKFB3. Among the highly deregulated genes, ADH1C showed a fold change of −6.669.

## 5 Conclusion

The study signifies the expression profile, survival analysis of glucose dysregulated genes, and their correlation with various clinico pathological features, protein -protein, gene -gene interaction, gene ontology and KEGG enrichment in breast cancer. The study reveals that glucose related genes are highly dysregulated in breast cancer. The highly dysregulated genes include ADH1C, ADH4, ALDH1A3, ALDOC, FBP1, PCK1, PFKFB1, PFKFB3. Deregulation of glucose related genes is linked with a poor prognosis in BC patients. Thus, targeting glucose related genes will be an effective treatment strategy for BC patients.

## Declaration of competing interest

The authors declare that they have no known competing financial interests or personal relationships that could have appeared to influence the work reported in this paper.
